# Single dose of a rVSV-based vaccine elicits complete protection against severe fever with thrombocytopenia syndrome virus

**DOI:** 10.1038/s41541-018-0096-y

**Published:** 2019-01-25

**Authors:** Fangfang Dong, Dandan Li, Dan Wen, Suhua Li, Chaoyue Zhao, Yue Qi, Rohit K. Jangra, Cuiping Wu, Dequan Xia, Xing Zhang, Fei Deng, Kartik Chandran, Zhen Zou, Fei Yuan, Aihua Zheng

**Affiliations:** 1 State Key Laboratory of Integrated Management of Pest Insects and Rodents, Institute of Zoology, Chinese Academy of Sciences, Beijing, China; 2 University of Chinese Academy of Sciences, Beijing, China; 3 Department of Microbiology and Immunology, Albert Einstein College of Medicine, Bronx, NY USA; 4 Department of Infectious Disease, Yidu Central Hospital of Weifang, Weifang, China; 5 State Key Laboratory of Virology, Wuhan Institute of Virology, Chinese Academy of Sciences, Wuhan, China; 6 CAS Key Laboratory of Pathogenic Microbiology and Immunology, Institute of Microbiology, Chinese Academy of Sciences, Beijing, China; 7 College of Life Science, Henan Normal University, Xinxiang, China

## Abstract

Severe fever with thrombocytopenia virus (SFTSV) is an emerging tick-borne phlebovirus that causes lethal human disease, for which there are no licensed antiviral vaccines or therapies. Herein, we developed a live attenuated recombinant vesicular stomatitis virus (rVSV)-based vaccine candidate expressing the SFTSV Gn/Gc glycoproteins (rVSV-SFTSV/AH12-GP). High titers of cross-protective, broadly neutralizing antibodies were elicited by a single dose of rVSV-SFTSV/AH12-GP in both immunocompetent and immunocompromised mice against multiple strains of SFTSV and the related but distinct phlebovirus Heartland virus (HRTV). Remarkably, complete protection against lethal challenge with SFTSV was conferred in young and old immunocompromised mice irrespective of any pre-existing vector-specific immunity. Collectively, these results suggest that a rVSV vector expressing SFTSV glycoproteins is a promising candidate vaccine against two emerging phleboviruses associated with severe human diseases.

## Emerging viruses: SFTSV vaccine

Severe Fever with Thrombocytopenia Syndrome virus (SFTSV) is an emerging East-Asian arbovirus with a high case fatality that lacks an effective vaccine. Aihua Zheng and colleagues at the Chinese Academy of Sciences generate a recombinant vesicular stomatitis-based vaccine vector expressing SFTSV glycoproteins that are the main targets of neutralizing antibodies. This attenuated rVSV-SFTSV vaccine elicits potent neutralizing antibodies in both young and aged mice but also in severely immunocompromised mice deficient in the type I interferon receptor (*Ifnar*^–/–^). Pre-existing vector-specific immunity did not compromise the generation of neutralizing antibodies. Importantly, rVSV-SFTSV could completely and durably protect *Ifnar*^–/–^ mice from lethal challenge with SFTSV. Serum transfer from immunized mice demonstrates protection is in large part mediated by antibodies. rVSV-SFTSV based approaches might therefore represent a promising vaccine candidate for a potentially important emerging virus.

## Introduction

Severe fever with thrombocytopenia virus (SFTSV) is an emerging tick-borne arbovirus first reported in East and Central China in 2009,^1^ and subsequently in Korea^2^ and Japan.^3^ A closely related virus, termed Heartland virus (HRTV), was identified and isolated from two patients in the United States in 2012.^4,5^ SFTSV infection causes fever, fatigue, and gastrointestinal disorders such as vomiting and diarrhea, along with leukocytopenia and thrombocytopenia.^1,6,7^ SFTSV is transmitted by tick bites and *Haemaphysalis Iongicornis* is the major vector.^8,9^ Humans appear to be an accidental host and play no role in the viral life cycle. Despite the severe disease caused by SFTSV and fatality rate of 2–30%, no vaccines or therapies are currently available.

SFTSV and HRTV are phleboviruses (Genus: *Phlebovirus*, Family: *Phenuiviridae*, Order: *Bunyavirales*) with enveloped, spherical- shaped virions of about 100 nm in diameter.^10,11^ Their viral genomes consist of three segments of ambisense or negative-sense RNAs: large (L), medium (M), and small (S). L encodes for the RNA-dependent RNA polymerase, M for the envelope Gn/Gc glycoproteins, and S for the nucleocapsid protein (NP) and the nonstructural protein.^12^ Gn/Gc forms non-covalent heterodimers that decorate the viral envelope and play a role in receptor binding and membrane fusion.^13–17^ Phylogenetic analysis suggests that SFTSV isolates cluster into the Chinese clade and Japanese clade, which is consistent with its geographic distribution.^18^ The Chinese clade can be further subdivided into clades 1–4.^19^ The Korean strains are clustered within the Japanese clade. Although HRTV is most closely related to SFTSV based on the phylogenetic analysis, there are about 27 and 38% differences in the amino acid sequences of the viral polymerase and nucleoprotein.^4^ The SFTSV Gn/Gc glycoproteins are also the primary targets of neutralizing antibodies.^17,20^ SFTSV neutralization antibodies from inoculated mice and rabbits cross-reacted with HRTV at a titer of 1:40 to 1:1280.^21^

Animal models are important tools for the study of viral pathogenesis and the development of vaccine and antiviral therapies. SFTSV infection in the C57/BL6 mice and rhesus macaques leads to viremia along with the hallmark clinical findings of leukocytopenia and thrombocytopenia, and only low titers of neutralizing antibodies are detected.^22,23^ In contrast to the mild symptoms in the immunocompetent animals, the interferon α/β receptor knockout (IFNAR^−/−^) mouse develops severe lymphocytopenia and thrombocytopenia, as seen in human SFTSV infection, and is therefore the only small-animal model currently available to evaluate the efficacy of vaccines and antiviral therapies.^21,24^ The IFNAR^−/−^ mouse has been widely used for evaluating antiviral vaccines, including those against dengue virus, Zika virus, and Crimean–Congo hemorrhagic fever virus.^25–28^

Vesicular stomatitis virus (VSV) is an enveloped negative-strand RNA virus of the family *Rhabdoviridae*. It has been developed as a very promising attenuated viral vaccine vector and has shown strong efficacy in eliciting neutralizing antibodies. Animals immunized with replication-competent recombinant VSV (rVSV)-based vaccine candidates are efficiently protected against lethal challenges with a variety of pathogens, such as Ebola virus, Marburg virus, Lassa virus, Hendra virus, Nipah virus, and Andes virus.^29–32^ Moreover, only a short and limited VSV viremia was detected in vaccine recipients in clinical trials, and these vectors were generally well tolerated.^33^ An rVSV virus expressing Ebola virus glycoprotein (rVSV-ZEBOV) was tested in a clinical phase 1/2 trial^34,35^ and the recent Ebola ça Suffit! trial in Guinea suggested that this vaccine is highly protective and effective against Ebola virus.^36^ In May 2017, Democratic Republic of the Congo approved the use of rVSV-ZEBOV vaccine, and it is on a path to FDA approval.^37^

Despite the wide distribution and high fatality caused by SFTSV and the recently discovered HRTV, vaccines against these pathogens are still unavailable.^38^ Here, we developed a replication-competent rVSV vector expressing SFTSV Gn/Gc as a live attenuated virus vaccine. We found that a single dose of rVSV vector expressing SFTSV or HRTV Gn/Gc elicited broad-spectrum neutralizing antibodies in both immunocompetent and immunocompromised IFNAR^−/−^ mice, and afforded 100% protection against high-dose SFTSV challenge. Our study leverages the rVSV platform to identify a promising candidate for the development into an SFTSV vaccine, and points to the utility of this platform for development of vaccine vectors against other virulent bunyaviruses.

## Results

### Construction and characterization of replication-competent rVSV vectors expressing SFTSV and HRTV glycoproteins

**Fig. 1:**
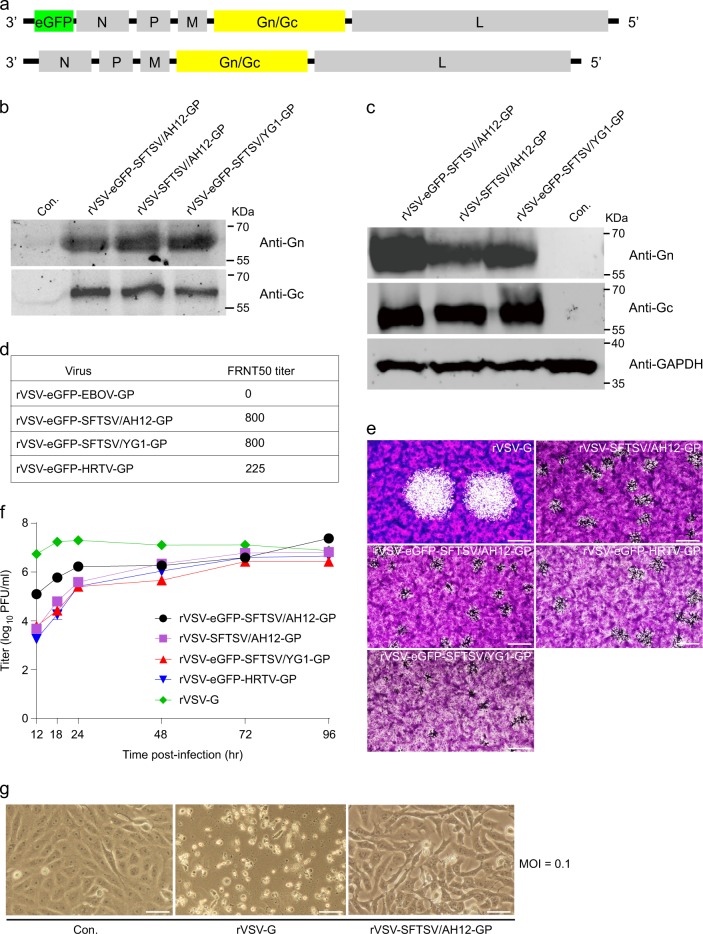
Characterization of the rVSV viruses. **a** Schematic diagrams show the organization of reporter and viral proteins in rVSV and rVSV-eGFP vectors. **b** Examining the incorporation of SFTSV Gn/Gc into the rVSV vectors. Rescued rVSV-SFTSV/AH12-GP, rVSV-eGFP-SFTSV/AH12-GP, and rVSV-eGFP-SFTSV/YG1-GP in the supernatant of 293 T cells were pelleted by ultracentrifugation and blotted with polyclonal antibodies against SFTSV Gn and Gc. Control (Con.) is the supernatant of non-transfected 293 T cells. Full scans of the blots are in Supplementary Fig 3. All blots derived from the same experiment and were processed in parallel. **c** Examining the expression of SFTSV Gn/Gc in rVSV-infected cells. Vero cells infected with rVSV-SFTSV/AH12-GP, rVSV-eGFP-SFTSV/AH12-GP, or rVSV-eGFP-SFTSV/YG1-GP were lysed and blotted as above. Control (Con.) is the noninfected cells. Full scans of the blots are in Supplementary Fig 3. All blots derived from the same experiment and were processed in parallel. **d** Neutralizing response of the rVSV-eGFPs to the serum from a convalescent SFTS patient as measured on Vero cells via an FRNT assay. **e** Plaque formation of rVSVs in Vero cells. Vero cells were infected with indicated viruses and stained by crystal violet 48 h (rVSV-G) or 96 h (all the other viruses) post infection. Scale bars indicate 500 μm. Data are representatives of three independent experiments. **f** Growth curves of rVSV viruses. Vero cells were infected by the rVSV viruses at an M.O.I. of 0.01 and virus titer in the supernatant was measured at the indicated time points post infection via a plaque assay on Vero cells. Each bar shows 95% cl. **g** Cytopathic effects of rVSV-SFTSV/AH12-GP. Vero cells were infected by the indicated viruses at an M.O.I. of 0.1 and bright-field images were taken 24 h post infection. Control (Con.) is the noninfected cells. Scale bars indicate 125 μm. Above data are representatives of three independent experiments

To develop rVSV-SFTSV vectors for evaluation of their vaccine potential, a series of rVSV plasmids were constructed.^14,39–41^ The human codon-optimized glycoprotein Gn/Gc ORF of the Chinese clade of SFTSV, AH12 strain (GenBank accession number ADZ04482.1)^42^ was cloned into a rVSVΔG vector as the vaccine candidate^43^ (designated as rVSV-SFTSV/AH12-GP). To evaluate the neutralizing activity, rVSVΔG-eGFP vector encoding an enhanced GFP reporter was modified to express human codon-optimized Gn/Gc glycoproteins of SFTSV AH12 strain, SFTSV YG1 strain (GenBank accession number BAN58185.1) representing the Japanese clade (98.5% identical with AH12 in amino acid sequence)^3^ or HRTV Missouri strain (GenBank accession number AFP33393.1, 62.0% identical with AH12 in amino acid sequence)^4^ (Fig. 1a). These strains were designated as rVSV-eGFP-SFTSV/AH12-GP, rVSV-eGFP-SFTSV/YG1-GP, and rVSV-eGFP-HRTV-GP, respectively. Additionally, rVSV-G, rVSV-eGFP-G (expressing the VSV G), rVSV-eGFP-EBOV-GP (expressing the Ebola virus GP),^41^ and rVSV-eGFP-HTNV-GP (expressing the Hantaan virus GP)^39^ were used as controls.

The rVSVs were recovered in the 293 T cells.^44^ The viral particles in the supernatant were pelleted by ultracentrifugation and incorporation of viral glycoproteins was verified by immunoblotting using polyclonal antibodies against SFTSV Gn and Gc.^17^ As shown in Fig. 1b,c both Gn and Gc were detected in rVSV-SFTSV/AH12-GP, rVSV-eGFP-SFTSV/AH12-GP, and rVSV-eGFP-SFTSV/YG1-GP virions, as well as in the virus-infected cells (Fig. S3). The rVSV-SFTSV/AH12-GP, rVSV-eGFP-SFTSV/AH12-GP, rVSV-eGFP-SFTSV/YG1-GP, and rVSV-eGFP-HRTV-GP were all passaged on Vero cells for four rounds. Viral RNAs were extracted for complete-genome sequencing and no mutations were detected, which suggest that these recombinant viruses are genetically stable. To evaluate whether the recombinantly expressed Gn/Gc proteins are folded properly and display major neutralizing epitopes as the authentic SFTSV, neutralization of rVSVs was tested against a serum from a convalescent SFTS patient via a focus reduction neutralization test (FRNT). The FRNT50 titers of both SFTSV AH12 and YG1 were about 800, while that of the HRTV was around 225 (Fig. 1d). As expected, no neutralizing activity was detected against rVSV-eGFP-EBOV-GP,^41^ the negative control (Fig. 1d). These results indicate that the SFTSV and HRTV Gn/Gc glycoproteins were properly incorporated onto the surface of rVSVs and displayed key neutralizing epitopes.

The plaque formation of rVSVs was tested in Vero cells. rVSV-G formed plaques with a diameter of ~1.2 mm at 48 h post infection, while rVSV-SFTSV/AH12-GP, rVSV-eGFP-SFTSV/AH12-GP, rVSV-eGFP-SFTSV/YG1-GP, and rVSV-eGFP-HRTV-GP developed very tiny plaques, which were barely visible, even at 96 h post infection (Fig. 1e). To investigate the viral growth in vitro, Vero cells were infected with the rVSVs at low M.O.I. The titer of rVSV-G in the supernatant reached about 2 × 10^7^ PFU/ml at 24 h post infection (Fig. 1f) and most of the rVSV-G-infected Vero cells were dead by that time (Fig. 1g). However, the titers of rVSV-SFTSV/AH12-GP, rVSV-eGFP-SFTSV/AH12-GP, rVSV-eGFP-SFTSV/YG1-GP, and rVSV-eGFP-HRTV-GP slowly increased and peaked at around 96 h post infection with mild cytopathic effects (Fig. 1f,g). These results suggest that the recombinant viruses were significantly attenuated as compared to the rVSV-G.

**Fig. 2:**
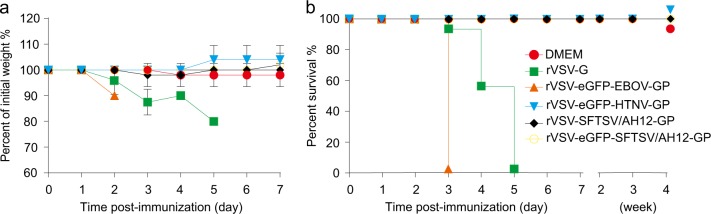
Virulence of rVSV viruses in IFNAR^−/−^ C57/BL6 mice. Groups of five mice, 6–8 weeks old, were inoculated (i.p.) with 2 × 10^4^ PFU of indicated viruses and then monitored for weight loss **a** and mortality **b**. Overlapped lines were indicated on the right end. Data are representatives of three independent experiments (error bars represent SD)

To formally test the attenuation of rVSVs in vivo, 6–8-week-old C57/BL6 mice were inoculated intraperitoneally (i.p.) with 2 × 10^4^ PFU of rVSV or rVSV-eGFP viruses and monitored for 1 week. None of the inoculated mice showed any clinical signs or weight loss. Next, we inoculated the immunocompromised interferon α/β receptor knockout (IFNAR^−/−^) C57/BL6 mice with rVSV and rVSV-eGFP and evaluated them for signs of morbidity and mortality as above. No significant weight loss and clinical signs were induced by rVSV-SFTSV/AH12-GP, rVSV-eGFP-SFTSV/AH12-GP, or rVSV-eGFP-HTNV-GP, while rVSV-G and rVSV-eGFP-EBOV-GP killed all the mice within 3–5 days (Fig. 2). Together, our results suggest that rVSV-SFTSV/AH12-GP was safe in both immunocompetent, as well as immunocompromised mice.

### Cross-reactive neutralizing antibodies elicited by rVSV-SFTSV/AH12-GP and rVSV-eGFP-HRTV-GP in immunocompetent and immunocompromised mice

**Fig. 3:**
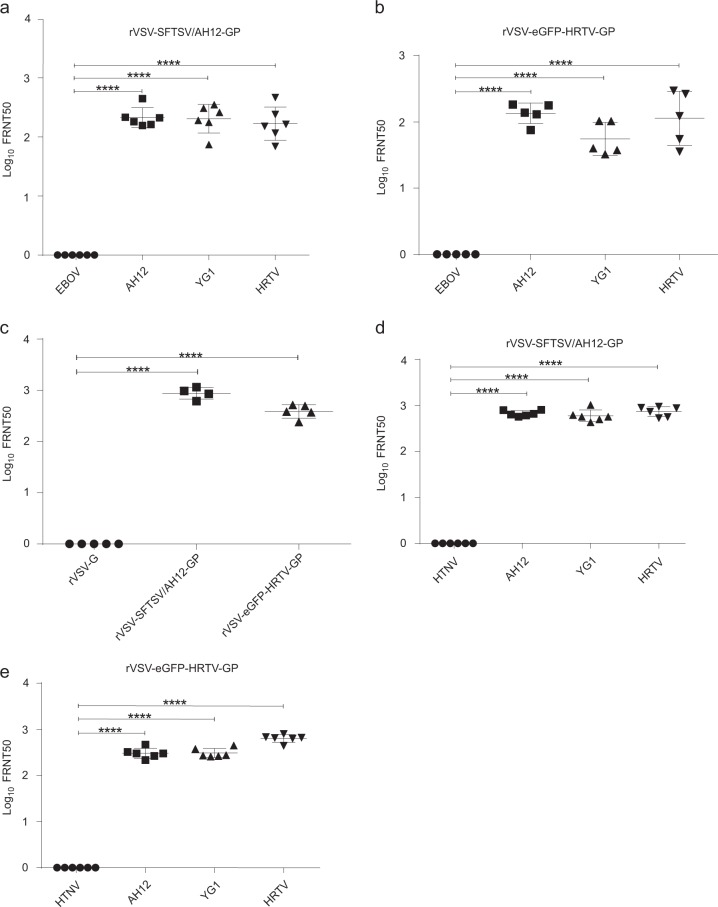
Broad-spectrum neutralizing antibodies elicited by rVSV-SFTSV/AH12-GP and rVSV-eGFP-HRTV-GP in immunocompetent and immunocompromised mouse models. **a**–**c** Groups of six or five C57/BL6 mice, 6–8 weeks old, were i.p. immunized with 2 × 10^4^ PFU of rVSV-SFTSV/AH12-GP **a** or rVSV-eGFP-HRTV-GP **b**. On day 30 post immunization, mouse sera were quantified for neutralization of rVSV-eGFP-SFTSV/AH12-GP (AH12), rVSV-eGFP-SFTSV/YG1-GP (YG1), rVSV-eGFP-HRTV-GP (HRTV), and control rVSV-eGFP-EBOV-GP (EBOV) via an FRNT assay. Each point represents one animal. **c** Sera from mice **a**, **b** immunized with rVSV-G (VSV), rVSV-SFTSV/AH12-GP (AH12), or rVSV-eGFP-HRTV-GP (HRTV) were also quantified for neutralization of SFTSV Wuhan (WH) strain via an FRNT assay. Each point represents one animal. Data are representative of three independent experiments (error bars represent SD). Significance was calculated using a multiple *t* test (****, *P*-value < 0.0001). **d**, **e** Six-to-eight-week-old IFNAR^−/−^ C57/BL6 mice were i.p. immunized with a single dose of 2 × 10^4^ PFU rVSV-SFTSV/AH12-GP **d** or rVSV-eGFP-HRTV-GP **e**. On day 30 post immunization, mouse sera were quantified for the neutralizing effect as above using rVSV-eGFP-HTNV-GP (HTNV) as control. Above data are representatives of three independent experiments and error bars indicate the SD. Significance was calculated using a multiple *t* test—one per row between control group and experimental groups (****P-value < 0.0001)

Next, we evaluated the immunogenicity of rVSV-SFTSV/AH12-GP in the wild-type and immunocompromised C57/BL6 mice.^22^ Six-to-eight-week-old C57/BL6 mice were i.p. immunized with a single dose of 2 × 10^4^ PFU of rVSV-SFTSV/AH12-GP or the control rVSV-G. On day 30 post immunization, mice were phlebotomized for neutralizing antibodies analysis using FRNT assay with rVSV-eGFPs. Vaccination with rVSV-SFTSV/AH12-GP elicited strong broad-spectrum neutralizing antibodies targeting the glycoproteins of SFTSV and HRTV with FRNT50 titers of 227, 229, and 201 for YG1, AH12, and HRTV, respectively, but none against the control EBOV glycoprotein (Fig. 3a). As expected, mice immunized with the negative control rVSV-G did not generate neutralization activity against rVSV-eGFPs expressing GPs of SFTSV or HRTV. To further test the cross-reactive immunogenicity between SFTSV and HRTV, we immunized C57/BL6 mice with rVSV expressing the glycoproteins of HRTV. Because our efforts to rescue the rVSV-HRTV-GP without the eGFP reporter did not pan out, we immunized C57/BL6 mice with rVSV-eGFP-HRTV-GP or rVSV-eGFP-G (as a negative control) at a single dose of 2 × 10^4^ PFU i.p. as mentioned above. rVSV-eGFP-HRTV-GP immunization also elicited broad-spectrum neutralizing antibodies with FRNT50 titers of 141, 64, and 155 for AH12, YG1, and HRTV, respectively (Fig. 3b). No detectable neutralizing activity against SFTSV and HRTV was detected in mice immunized with rVSV-eGFP-G. The neutralization results were also confirmed using authentic SFTSV field strain Wuhan, which is 99.3% identical to the AH12 strain in the amino acid sequence of GP,^45^ showing a FRNT50 titer of 899 by rVSV-SFTSV/AH12-GP and 402 by rVSV-eGFP-HRTV-GP (Fig. 3c). These results suggest that the SFTSV and HRTV glycoproteins expressed by rVSV vector could generate cross-reactive humoral immunity in the immunocompetent mouse model.

As IFNAR^−/−^ mice are the only small-animal model to evaluate SFTSV vaccine efficacy, immunocompromised IFNAR^−/−^ C57/BL6 mice were immunized with rVSV-SFTSV/AH12-GP or rVSV-eGFP-HRTV-GP as above. rVSV-SFTSV/AH12-GP induced broad-spectrum neutralizing antibody against rVSV-eGFPs expressing GP of AH12, YG1, and HRTV with titers of 682, 623, and 802, respectively (Fig. 3d). The corresponding titers of neutralizing antibodies elicited by rVSV-eGFP-HRTV-GP were 312, 316, and 644 against the same set rVSV-eGFPs (Fig. 3e). In both cases, no neutralizing activity was seen against rVSV-eGFP expressing HTNV glycoproteins. FRNT50 titers elicited by both rVSV-SFTSV/AH12-GP and rVSV-eGFP-HRTV-GP against the authentic SFTSV field strain were higher than 1600. These data demonstrate that rVSV-SFTSV/AH12-GP can induce robust, cross-reactive humoral immune response in both immunocompetent and immunocompromised mouse models.

### Vaccination with both rVSV-SFTSV/AH12-GP and rVSV-eGFP-HRTV-GP confers complete cross-protection against lethal SFTSV challenge

**Fig. 4:**
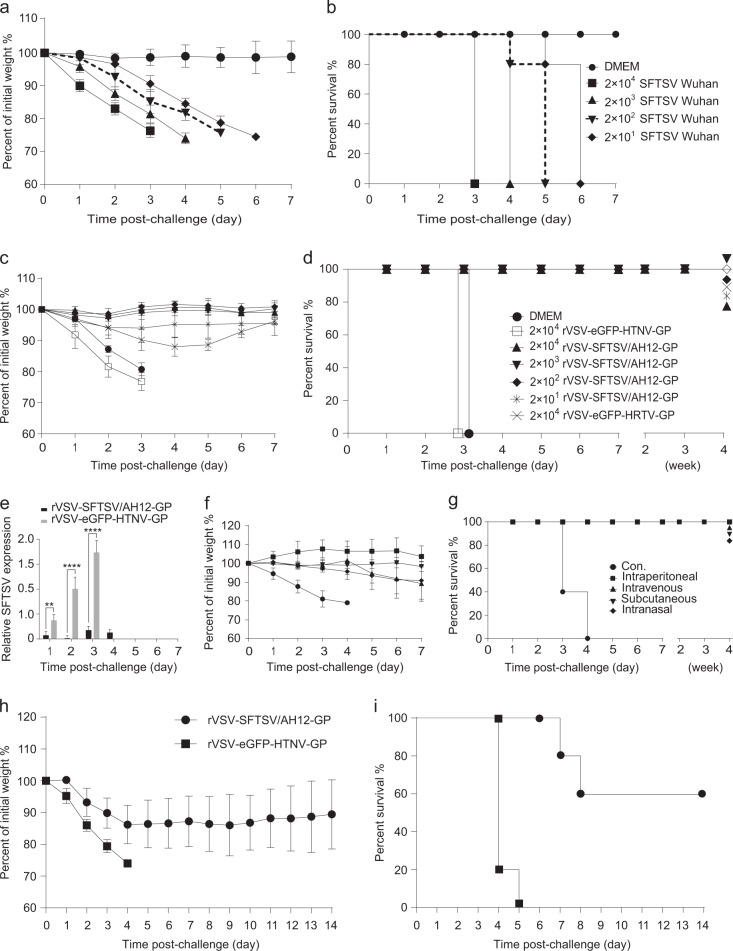
A single dose of rVSV-SFTSV/AH12-GP or rVSV-eGFP-HRTV-GP elicits complete protection against lethal dose challenge with SFTSV Wuhan strain in IFNAR^−/−^ C57/BL6 mice. **a**, **b** Susceptibility of IFNAR^−/−^ mice to SFTSV. Six-to-eight-week-old mice (*n* = 5/group), were i.p. inoculated with 2 × 10^1^ to 2 × 10^4^ FFU of SFTSV Wuhan strain and then monitored for weight loss **a** and mortality **b**. DMEM was used as control. Data are representative of three independent experiments (error bars represent SD). **c**, **d** IFNAR^−/−^ mice (*n* = 5/group), were immunized with the indicated amount (PFU) of rVSV-SFTSV/AH12-GP, rVSV-eGFP-HRTV-GP, rVSV-eGFP-HTNV-GP, or DMEM control and then challenged with 2 × 10^4^ FFU of SFTSV Wuhan strain at 30 days post immunization. Weight loss **c** and mortality **d** were monitored for indicated time. Overlapped lines were indicated on the right end. **e**. Mice immunized with 2 × 10^4^ PFU of rVSV-SFTSV/AH12-GP or rVSV-eGFP-HTNV-GP were tested for viremia after SFTSV challenge as above. Above data are representatives of three independent experiments (error bars represent SD). Significance was calculated using a multiple *t* test—one per row between control group and experimental groups (*P-value < 0.05; **P-value < 0.01; ****P-value < 0.0001). **f**, **g** Immunization route does not affect the efficacy of rVSV-SFTSV/AH12-GP. IFNAR^−/−^ mice (*n* = 5/group), were immunized with 1 × 10^4^ PFU of rVSV-SFTSV/AH12-GP by intraperitoneal, intravenous, subcutaneous, and intranasal route, and then i.p. challenged with 2 × 10^4^ FFU of SFTSV Wuhan strain at 30 days post immunization. Control group was not immunized (Con.). Weight loss **f** and mortality **g** were monitored for indicated time. Overlapped lines are indicated on the right end. Above data are representatives of two independent experiments (error bars represent SD). **h**, **i** Passive transfer of sera from rVSV-SFTSV/AH12-GP immunized mice confers protection in naive mice. Sera were prepared from IFNAR^−/−^ mice immunized with 2 × 10^4^ PFU of rVSV-SFTSV/AH12-GP or rVSV-eGFP-HTNV-GP at 30 days post immunization. Five serum samples of each group were randomly injected into five naive IFNAR^−/−^ mice (i.p.); 24 h later, recipient mice were challenged with 2 × 10^3^ FFU of SFTSV Wuhan strain (i.p.) and monitored for weight loss **h** and mortality **i**. Above data are representatives of three independent experiments

Several reports have demonstrated that SFTSV infection in immunocompromised interferon α/β receptor knockout (IFNAR^−/−^) C57/BL6 mice is lethal,^21,24^ making it an ideal model for the assessment of the protective efficacy of vaccine candidates. We first tested the susceptibility of IFNAR^−/−^ mice to SFTSV by inoculating 6–8-week-old male and female animals with 2 × 10^1^ to 2 × 10^4^ FFU of SFTSV Wuhan strain (i.p.). Upon infection, the mice developed clinical signs such as hunched posture, ruffled fur, and weight loss. The infected animals were euthanized when they lost >25% of their body weight or had bilateral hind limb paralysis (Fig. 4a). As shown in Fig. 4b, all the animals died at 3–6 days post infection, even the ones inoculated with 20 FFU of SFTSV suggesting that IFNAR^−/−^ mice were highly susceptible to SFTSV infection with an LD50 of less than 20 FFU.

To investigate the protection efficacy of the vaccine candidate, 6–8-week-old IFNAR^−/−^ mice were immunized with 2 × 10^4^ to 2 × 10^1^ PFU rVSV-SFTSV/AH12-GP, i.p. in 10-fold serial dilutions or 2 × 10^4^ PFU of rVSV-eGFP-HRTV-GP, DMEM and 2 × 10^4^ PFU of rVSV-eGFP-HTNV-GP as negative controls for immunizations. No obvious weight loss was detected in the animals during the monitoring period of 1 week (Fig. S1). The vaccinated animals were subsequently challenged 30 days post immunization with 2 × 10^4^ FFU (higher than 10^3^ LD50) of SFTSV Wuhan strain (i.p.) and the animals were observed for development of clinical signs and weight loss. The control groups lost weight quickly and died within 3 days post challenge. All the mice immunized with either rVSV-SFTSV/AH12-GP or rVSV-eGFP-HRTV-GP survived for at least 1 month. Mice immunized with rVSV-eGFP-HRTV-GP showed a maximum of 10% weight loss at day 3 to day 5 and recovered by day 6, while no weight loss was observed in the rVSV-SFTSV/AH12-GP-immunized mice (Fig. 4c,d). SFTSV viremia was detected in the blood of mice immunized with the negative control rVSV-eGFP-HTNV-GP at 1 day post challenge and it continually increased until death. Remarkably, only very low viremia was detected on day 3 to day 4 post challenge in mice immunized with rVSV-SFTSV/AH12-GP (Fig. 4e). These results suggested that the rVSV-SFTSV/AH12-GP and rVSV-eGFP-HRTV-GP were highly protective against SFTSV challenge.

To test the effect of different immunization routes on the efficacy, groups of five IFNAR^−/−^ mice were immunized by intraperitoneal, intravenous, subcutaneous, and intranasal routes, respectively with 1 × 10^4^ PFU of rVSV-SFTSV/AH12-GP in different volumes. No weight loss was detected up to 1 week after immunization (Fig. S2). After 28 days, groups of mice were challenged with 2 × 10^4^ FFU of SFTSV Wuhan strain, i.p.. All the rVSV-SFTSV/AH12-GP immunized mice survived without any obvious clinical signs or significant weight loss, while the control (untreated) group died on day 3–4 post challenge (Fig. 4f,g). These results suggest that rVSV-SFTSV/AH12-GP can be delivered by many routes without affecting its protective efficacy.

Our study demonstrates strong humoral immunity elicited by rVSV-SFTSV/AH12-GP in mice. To further test the effect of humoral immunity on protection, sera from the immunized mice were passively transferred into naive mice. IFNAR^−/−^ mice were immunized with 2 × 10^4^ PFU rVSV-SFTSV/AH12-GP or control rVSV-eGFP-HTNV-GP (five mice/group; i.p.) and sera were prepared 30 days later. Groups of five IFNAR^−/−^ mice were injected with 200 ul of immunized sera and subsequently challenged with 2 × 10^3^ FFU (higher than 10^2^ LD50) of SFTSV Wuhan strain 24 h later. All the mice that received sera from rVSV-eGFP-HTNV-GP-immunized mice showed rapid weight loss and died on day 4–5 post challenge. However, the group of mice that received sera from rVSV-SFTSV/AH12-GP-immunized mice exhibited slower weight loss and 60% survived the challenge, while the other 40% died during day 7–8 (Fig. 4h,i). These results further suggest that rVSV-SFTSV/AH12-GP could elicit strong humoral immunity in mice, which plays a key role in protection against SFTSV infection.

### rVSV-SFTSV/AH12-GP elicits complete protection in mice with pre-existing anti-VSV immunity

**Fig. 5:**
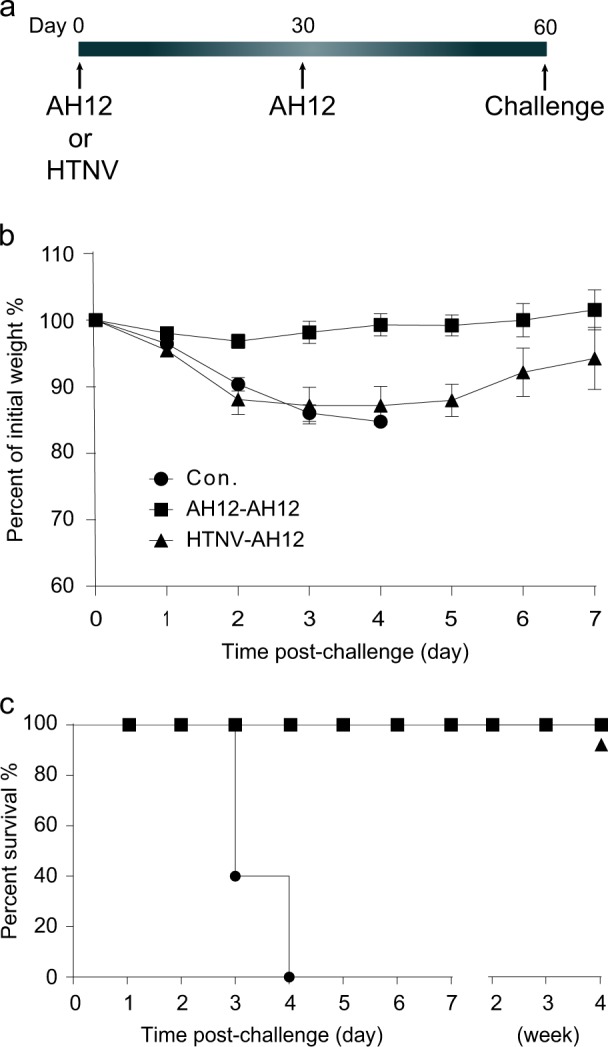
Full protection was conferred by a single dose of rVSV-SFTSV/AH12-GP in mice pre-immunized with rVSV vector. Six-to-eight-week-old IFNAR^−/−^ mice were immunized with 2 × 10^4^ PFU of rVSV-SFTSV/AH12-GP or rVSV-eGFP-HTNV-GP. Thirty days later, all mice were further immunized with 2 × 10^4^ PFU of rVSV-SFTSV/AH12-GP and challenged with 2 × 10^4^ FFU of SFTSV Wuhan strain on day 60, using naive mice at the same age as negative control **a**. All the above injections were i.p. route. Weight loss **b** and mortality **c** were monitored for indicated time. Overlapped lines are indicated on the right end. Above data are representatives of three independent experiments (error bars represent SD)

Pre-existing vector-specific immunity might decrease or nullify the efficacy of vector-based vaccine. To test the effect of pre-existing anti-VSV immunity on the efficacy of rVSV-SFTSV/AH12-GP, 6–8-week-old IFNAR^−/−^ mice were pre-immunized with 2 × 10^4^ PFU, i.p., of rVSV-SFTSV/AH12-GP or rVSV-eGFP-HTNV-GP, respectively. Thirty days later, all animals were vaccinated with 2 × 10^4^ PFU rVSV-SFTSV/AH12-GP, i.p.. After another 30 days, all the animals were challenged with 2 × 10^4^ FFU of SFTSV Wuhan strain, i.p. (Fig. 5a). All the rVSV-SFTSV/AH12-GP twice immunized mice survived without any obvious disease and weight loss. Animals pre-immunized with rVSV-eGFP-HTNV-GP showed about 10% weight loss and recovered by day 7 and survived for at least 1 month, while the untreated, control animals died on day 3–4 post challenge (Fig. 5b,c). As shown previously for rVSV-based Lassa fever virus and Ebola virus vaccines,^29^ the efficacy of rVSV-SFTSV/AH12-GP was not affected by pre-existing vector-specific immunity.

### rVSV-SFTSV/AH12-GP protects aged IFNAR^−/−^ mice from lethal SFTSV infection

**Fig. 6:**
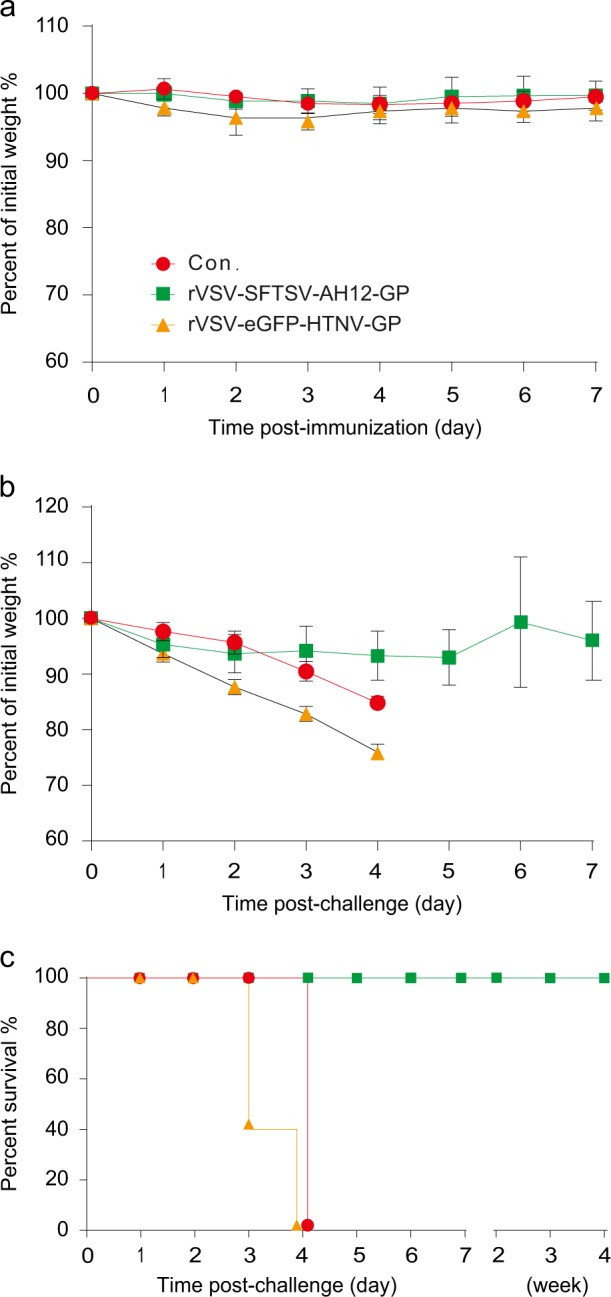
Old IFNAR^−/−^ C57/BL6 mice were completely protected by rVSV-SFTSV/AH12-GP against lethal SFTSV challenge. Eight-month-old IFNAR^−/−^ C57/BL6 mice were immunized with 2 × 10^4^ PFU of rVSV-SFTSV/AH12-GP or rVSV-eGFP-HTNV-GP using untreated mice as control and weight change was monitored for 1 week **a**. On day 30 post immunization, all mice were i.p. challenged with 2 × 10^4^ FFU of SFTSV Wuhan strain. Weight loss **b** and mortality **c** were monitored for indicated time. Above data are representatives of three independent experiments (error bars represent SD)

Reports from hospitals suggest that most of the SFTSV patients are elderly with a median age of 61 years old.^1^ To test the efficacy of vaccine in the target elderly population, 8–9-month-old IFNAR^−/−^ mice were immunized with 2 × 10^4^ PFU of rVSV-SFTSV/AH12-GP or rVSV-eGFP-HTNV-GP, i.p.. No obvious weight loss was detected in the animals during the monitoring period of 1 week (Fig. 6a). After 28 days, groups of mice were challenged with 2 × 10^4^ FFU of SFTSV Wuhan strain, i.p.. Animals that were untreated or inoculated with rVSV-eGFP-HTNV-GP lost weight quickly and died within 3–4 days post-SFTSV challenge. On the other hand, in mice immunized with rVSV-SFTSV/AH12-GP, no significant weight loss was detected and all the mice survived (Fig. 6b,c). These results suggest that rVSV-SFTSV/AH12-GP is effective in aged mice and is a promising candidate for further development given that the elderly patients are at high risk of mortality upon SFTSV infection.

## Discussion

Viruses of the order of *Bunyavirales*, include many highly pathogenic viruses such as hantaviruses (e.g., Hantaan virus), Crimean–Congo hemorrhagic fever, Rift Valley fever virus, and SFTSV. Despite considerable research efforts, there are still no safe and effective vaccines available for any of the bunyaviruses. Some live-attenuated and inactivated virus vaccines against hantaviruses are licensed only in East-Asian countries.^46^ The rVSV-based Andes virus vaccine is still under pre-clinical development.^31^ The live attenuated Rift Valley fever vaccine, MP-12, finished Phase 1/2 clinical trial.^47,48^ There is an urgent need for the development of an effective SFTSV vaccine for its control. Here, we have demonstrated that an rVSV vector expressing the SFTSV glycoproteins can confer strong broad-spectrum neutralizing responses against multiple SFTSV strains, as well as HRTV by single-dose immunization in both immunocompetent and immunocompromised mice. Importantly, complete cross-protection between SFTSV and HRTV was conferred in the immunocompromised lethal SFTSV mouse model. Significantly, old mice and mice with pre-existing vector-specific immunity were also fully protected by single-dose vaccination.

Safety is a major concern for live-virus vaccines. In our study, rVSV-SFTSV/AH12-GP was highly attenuated as demonstrated by its very low cytopathic effect in cell culture compared with rVSVs expressing glycoprotein of VSV or EBOV, which killed most cells in 1 or 2 days. The immunocompromised IFNAR^−/−^ mouse immunized with 2 × 10^4^ PFU of rVSV-SFTSV/AH12-GP showed neither weight loss nor any clinical signs of illness, while all the mice immunized with rVSVs expressing glycoprotein of VSV or EBOV died within 5 or 3 days, respectively. In addition, a single dose of 2 × 10^1^ PFU elicited complete protection against more than 10^3^ LD50 SFTSV challenge. These results suggest that rVSV-SFTSV/AH12-GP is a promising vaccine candidate with high safety.

Furthermore, rVSV-SFTSV/AH12-GP could also elicit broad-spectrum humoral immunity against both SFTSV and HRTV glycoproteins even though they are only 62% identical. To further confirm cross-reactivity, we immunized mice with rVSV expressing HRTV glycoproteins. As rVSV-HRTV-GP could not be rescued, we immunized mice with rVSV-eGFP-HRTV-GP carrying the eGFP reporter using rVSV-eGFP-HTNV-GP with the same eGFP reporter as the negative control vector. Our results indicated that eGFP had no obvious effect on the immunogenicity of the resulting vaccine vector and rVSV-eGFP-HRTV-GP generated neutralizing antibodies that were comparable to rVSV-SFTSV/AH12-GP. No neutralization was detected against HTNV, which belongs to the *Hantaviridae* family. We speculate that the moderate similarity of the Gn/Gc glycoproteins leads to the cross-reactive neutralizing and protection between HRTV and SFTSV. Consistent with our results, Matsuno et al. reported that SFTSV neutralization antibodies from inoculated mice and rabbits cross-reacted with HRTV at a titer of 1:40 to 1:1280.^21^ We observed in Fig. 4c that rVSV-eGFP-HRTV-GP-vaccinated mice showed slight weight loss after SFTSV challenge, while no weight loss was detected in groups vaccinated with rVSV-SFTSV/AH12-GP. However, rVSV-eGFP-HTNV-GP, encoding the glycoprotein of Hantaan virus which only shares 10% identical positions with SFTSV, did not protect mice against SFTSV challenge.

Previous reports have indicated that rVSV can induce strong humoral and cellular immune responses. Clinical research studies reveal that SFTSV infection induces moderate humoral immune response in patients with neutralizing antibody titer ranging from 80 to 640. However, only low titers less than 100 were detected in C57/BL6 mouse and Rhesus macaque animal models infected with SFTSV.^23^ In the present study, high titers of neutralizing antibody were generated by glycoproteins presented by rVSV vector with FRNT50 titer of 899 and more than 1600 in WT and IFNAR^−/−^ C57/BL6 mice, respectively. Mice immunized by intraperitoneal, intravenous, subcutaneous, and intranasal route show no difference in protection against lethal SFTSV challenge, which suggests that this rVSV-based SFTSV vaccine would be easy to inoculate.

Pre-existing vector-specific immunity is thought to attenuate the immune response of some vector-based vaccines. However, rVSV-based vaccines encode and express the glycoproteins of foreign pathogens without its own glycoproteins, suggesting that the pre-existing immunity would not interfere with their efficacy. Sequential immunization of macaques with rVSV-based vaccines for Lassa fever and Ebola virus suggests that rVSV platform does not have the pre-existing immunity issue.^29^ In this report, we pre-immunized IFNAR^−/−^ C57/BL6 mice with rVSV vector expressing glycoprotein of HTNV 30 days before vaccination with rVSV-SFTSV/AH12-GP. All the animals were fully protected from lethal SFTSV challenge, which suggests that pre-existing immunity did not affect the efficacy of rVSV-SFTSV/AH12-GP.

Another major concern about the usage of SFTSV vaccine is the age of target vaccination population because reports from hospitals reveal that the medium age of the patient is 61. Aging-related immunosenescence will attenuate the efficacy of a vaccine. For the WT C57/BL6 mice, 18–24 months old is considered as old. However, the IFNAR^−/−^ C57/BL6 mice have a shorter life span and tend to die naturally around 12 months old. So, we chose 8–9-month-old IFNAR^−/−^ mice to evaluate the efficacy of rVSV-SFTSV/AH12-GP in the aged population. Remarkably, all the old mice were fully protected by a single dose of rVSV-SFTSV/AH12-GP against lethal SFTSV challenge. Our results suggest that the rVSV-SFTSV/AH12-GP vaccine candidate could be applied across a broad age range.

In summary, we found that Gn/Gc is an effective immunogen for SFTSV vaccine and rVSV live-attenuated vector is a promising vaccine platform. rVSV-SFTSV/AH12-GP could elicit robust and cross-reactive protection in broad-age range population with no interference from pre-existing VSV-specific immunity, which is promising for further clinical development.

## Methods

### Ethics statement

All animal studies were carried out in strict accordance with the recommendations in the Guide for the Care and Use of Laboratory Animals of the Ministry of Science and Technology of the People’s Republic of China. The protocols for animal studies were approved by the Committee on the Ethics of Animal Experiments of the Institute of Zoology, Chinese Academy of Sciences (Approval number: IOZ20170019). Studies with SFTSV were conducted under biosafety level 2 and animal biosafety level 3 containment. The virus challenge experiments were performed in a biosafety level 3 facility.

One human serum sample was obtained from Yidu Central Hospital of Weifang, Weifang, China. The studies and protocols were approved by the Committee on the Ethics of Human of the Yidu Central Hospital of Weifang. Written informed consent was obtained from the patient before enrollment in the study.

### Viruses, antibodies, and cells

SFTSV Wuhan strain (GenBank accession numbers: S, KU361341.1; M, KU361342.1; L, KU361343.1) and rabbit anti-SFTSV-NP polyclonal antibody were prepared in Wuhan Institute of Virology, Chinese Academy of Sciences.^45,49^ Polyclonal antibodies against SFTSV Gn and Gc were gifts from Dr. George F. Gao and Dr. Yan Wu, from the Institute of Microbiology, CAS.^17,50^ BHK-21 cells (baby hamster kidney), Vero cells (African green monkey kidney epithelial cells), and Huh7.5 cells (human hepatocarcinoma cells) were obtained from American Type Culture Collection (ATCC) and maintained in Dulbecco’s modified Eagle’s medium (DMEM, Hyclone, US) supplemented with 10% FBS and L-glutamine at 37 °C with 5% CO_2_.

### Construction and rescue of rVSV viruses

The rVSV plasmid was do novo synthesized following the original VSV reverse genetics system designed by Dr. Sean Whelan.^43^ Briefly, a lab-adaptive VSV Indiana strain was synthesized using KF935251.1 (GenBank sequence accession number) as the template by Genewiz Suzhou. An *MluI* site at nt3075 (KF935251.1 nucleotide number) and a *NotI* site at nt4621 were inserted flanking the G-protein-encoding region. rVSV-G vector was constructed by cloning the VSV sequence into a pGEM vector with a T7 promoter in front of the 3’ terminus and hepatitis delta virus (HDV) ribozyme terminal site following the 5’ end. The eGFP reporter was further added at nt62 to make rVSV-eGFP-G. The rVSV-eGFP-G and rVSV-G were used as backbone for the construction of all the other rVSVs. The humanized glycoprotein Gn/Gc sequences of SFTSV AH12 strain (GenBank accession number ADZ04482.1),^42^ SFTSV YG1 stain (GenBank accession number BAN58185.1),^3^ and HRTV Missouri strain (GenBank accession number AFP33393.1)^4^ were synthesized by Genewiz Suzhou and inserted between *MluI* and *NotI* restriction sites into the rVSVΔG vector or rVSVΔG-eGFP vector.^43,44^ rVSVs were rescued by co-transfecting the rVSV plasmid with plasmids expressing the *T7* polymerase and *N, P, M, G, L* of VSV into 293 T cells using calcium phosphate.^40^ Viruses in the supernatant were collected when the cytopathogenic effect was recognizable and further amplified in Vero cells for one passage as a stock.^40^ The identities of the rVSVs were verified by sequencing the glycoprotein region in each vector (Supplementary Table 1). The incorporation of glycoproteins Gn/Gc was verified by immunoblotting using polyclonal antibodies against SFTSV Gn and Gc.

### Experimental animals and immunization

Specific-pathogen-free C57/BL6 WT mice were purchased from Beijing Vital River Laboratory Animal Technology (licensed by Charles River). IFNα/β receptor knockout C57/BL6 mice (IFNAR^−/−^) were purchased from the Institute of Laboratory Animal Science, Chinese Academy of Medical Science and Peking Union Medical College and breed in the Institute of Zoology, CAS. Following acclimation, 6–8-week-old male and female WT or IFNAR^−/−^ mice (*n* = 5 per group) were immunized with a single dose of rVSV viruses expressing various viral glycoproteins. The volume applied as follows: for the intranasal route, 30 ul was split between both nostrils; for the i.p. route, 100 ul was split between two sites; for the subcutaneous route, 30 ul was administered in two footpads; and, for the intravenous route, 100 ul was applied in the tail vein. Body weight and clinical symptoms were observed for 7 days post immunization. IFNAR^−/−^ mice were further challenged with 2 × 10^4^ FFU of SFTSV Wuhan strain (two sites i.p., 100 ul each) at 30 days post immunization and monitored for weight loss and clinical symptoms. Mice were assigned a clinical score of increasing severity: 1, hunched posture; 2, ruffled fur; 3, hind limb paresis; 4, bilateral hind limb paralysis; 5, moribund. Mice with score 4 or a weight loss of more than 25% were euthanized. In this report, after the immunization or challenge of mice, the weight loss was monitored for 1 week and mortality was monitored for at least one extra week.

For the old mice immunization experiment, groups of six 8–9-month-old male and female IFNAR^−/−^ C57/BL6 mice were i.p. inoculated and evaluated using the same procedure as the young mice. For the pre-existing immunity experiments, groups of five 6–8-week-old IFNAR^−/−^ mice were i.p. pre-immunized with 2 × 10^4^ PFU of rVSV-SFTSV/AH12-GP or rVSV-eGFP-HTNV-GP, respectively. Thirty days later, all animals were i.p. vaccinated with 2 × 10^4^ PFU rVSV-SFTSV/AH12-GP. After another 30 days, groups of mice were i.p. challenged with 2 × 10^4^ FFU of SFTSV Wuhan strain.

For the passive serum transfer experiment, sera were prepared from IFNAR^−/−^ C57/BL6 mice immunized with 2 × 10^4^ PFU of rVSV-SFTSV/AH12-GP or negative control rVSV-eGFP-HTNV-GP at 30 days post immunization. Sera from five immunized mice were randomly injected into five 6–8-week-old naive mice at a dose of 200 ul each (i.p.). Twenty-four hours later, the recipient mice were challenged with 2 × 10^3^ FFU of SFTSV Wuhan strain as described above and body weight was monitored for 2 weeks.

### Virus titration and neutralization test

Focus-forming assay was performed in Huh7.5 cells to titrate the viral titers. Cells were plated in triplicates 24 h before infection in 96-well plates at 1.5 × 10^4^ cells per well in DMEM supplemented with 10% FBS. The virus samples were diluted 10-fold in DMEM with 2% FBS. After removal of medium, the cells were incubated with the 10-fold diluted viral solution at 37 °C. Three to four hours later, the cells were washed once and incubated with DMEM plus 10% FBS and 20 mM NH_4_Cl at 37 °C. For the rVSV-eGFPs, 20 h post infection, the virus titers were examined under a fluorescent microscope and calculated by Reed–Muench method. For the SFTSV Wuhan strain, 2 days post infection, the cells were fixed with cold methanol and stained using a rabbit anti-SFTSV-NP polyclonal antibody at 1:700 dilution and Alexa 488-labeled goat anti-rabbit IgG at 1:700 dilution.

For the FRNT assay, 100 FFU of SFTSV Wuhan strain were incubated with heat-inactivated sera diluted from 1:25 to 1:1600 by twofold serial dilution at RT for 30 min and then layered onto the cells in 384-well plates. The titers were assayed as described above and calculated by Reed–Muench method. Titers lower than 1:25 were designated as 0.

Plaque-forming assay was performed in BHK-21 cells to titrate the viral titers. BHK-21 cells were plated 24 h before infection in the 24-well plates at 3.0 × 10^4^ cells per well in triplicates. The virus samples were diluted 10-fold in DMEM with 2% FBS. After removal of medium, the cells were incubated with the 10-fold diluted viral solution at 37 °C. Three to four hours later, the cells were washed once and incubated with DMEM plus 10% FBS and 2% CMC-Hanks. The cells were incubated flat at 28 °C to allow plaques to form. The medium was aspirated and the cells were stained with crystal violet. The titers were calculated by Reed–Muench method.

### RNA isolation and real-time PCR

Blood samples were collected from mouse tail tips and total RNA was extracted by the TGuide cell/tissue/plant RNA kit (Tiangen, China). Samples were analyzed using a One Step SYBR PrimerScript reverse transcription (RT)-PCR kit (TaKaRa) on Applied Biosystems QuantStudio. Each sample was measured by triplicate in three independent experiments. β-actin transcript expression was used as an internal control for quantification. The relative expression levels were calculated using standard ΔΔCt method. Primer pairs were listed as follows: for the reference gene of β-actin: forward, GGCTGTATTCCCCTCCATCG; reverse, CCAGTTGGTAACAATGCCATGT. For SFTSV Wuhan strain: forward, ATGGATAGCAGCGTCTCATCAAATC; reverse, TGAGCGCACTGTATGAGGTAGGTAA (Supplementary Table 1). Real-time PCR reactions were initiated at 42 °C for 5 min, incubated at 95 °C for 10 s, and then followed by 40 cycles of 95 °C for 5 s and 60 °C for 20 s.

## Supplementary information

Supplementary information

## Data Availability

All data are fully available without restriction.

## Supplementary information

**Supplementary information** accompanies the paper on the *npj Vaccines* website (10.1038/s41541-018-0096-y).
