# Supplementary material for: Single dose of a rVSV-based vaccine elicits complete protection against severe fever with thrombocytopenia syndrome virus
Source: NPJ Vaccines. 2019 Jan 25;4:5. doi: 10.1038/s41541-018-0096-y (PMC6347601; doi:10.1038/s41541-018-0096-y)
Supplement: Supplementary file 1 — Supplementary information [file 41541_2018_96_MOESM1_ESM.pdf]

Figure S1

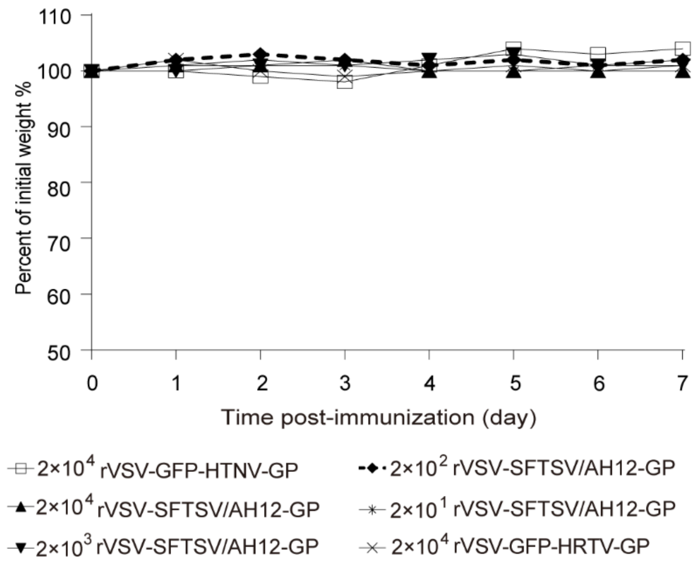

Supplementary figure 1. IFNAR$^{-/-}$ C57/BL6 mice do not show weight loss after immunization with rVSV-SFTSV/AH12-GP, rVSV-eGFP-HRTV-GP, or rVSV-eGFP-HTNV-GP.

Six to eight-weeks old IFNAR$^{-/-}$ mice (n=6/group), were immunized with indicated amount of rVSV viruses (i.p) and monitored for weight loss for one week. Data are representative of three independent experiments (error bars represent SD).

Figure S2

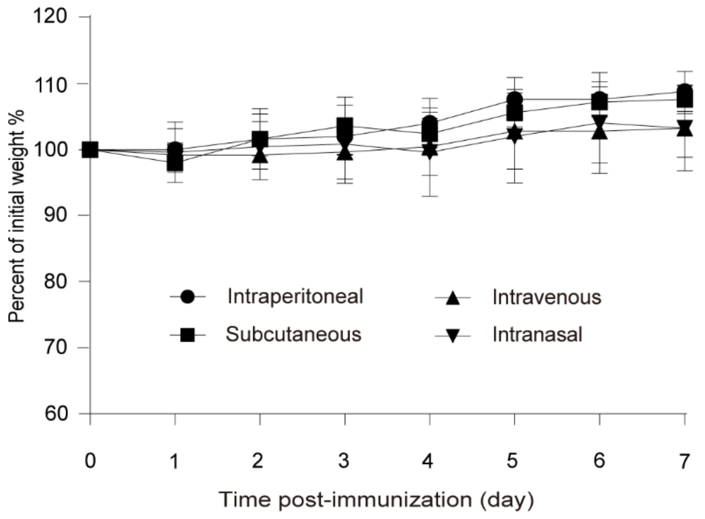

Supplementary figure 2. IFNAR$^{-/-}$ C57/BL6 mice do not show weight loss after immunization with rVSV-SFTSV/AH12-GP by different routes.

Six to eight weeks old IFNAR$^{-/-}$ mice (n=5/group), were immunized with 1x10^{4} PFU of rVSV-SFTSV/AH12-GP by intraperitoneal, intravenous, subcutaneous and intranasal route respectively, and then monitored for weight loss for a week. Data are representative of two independent experiments (error bars represent SD).

Figure S3

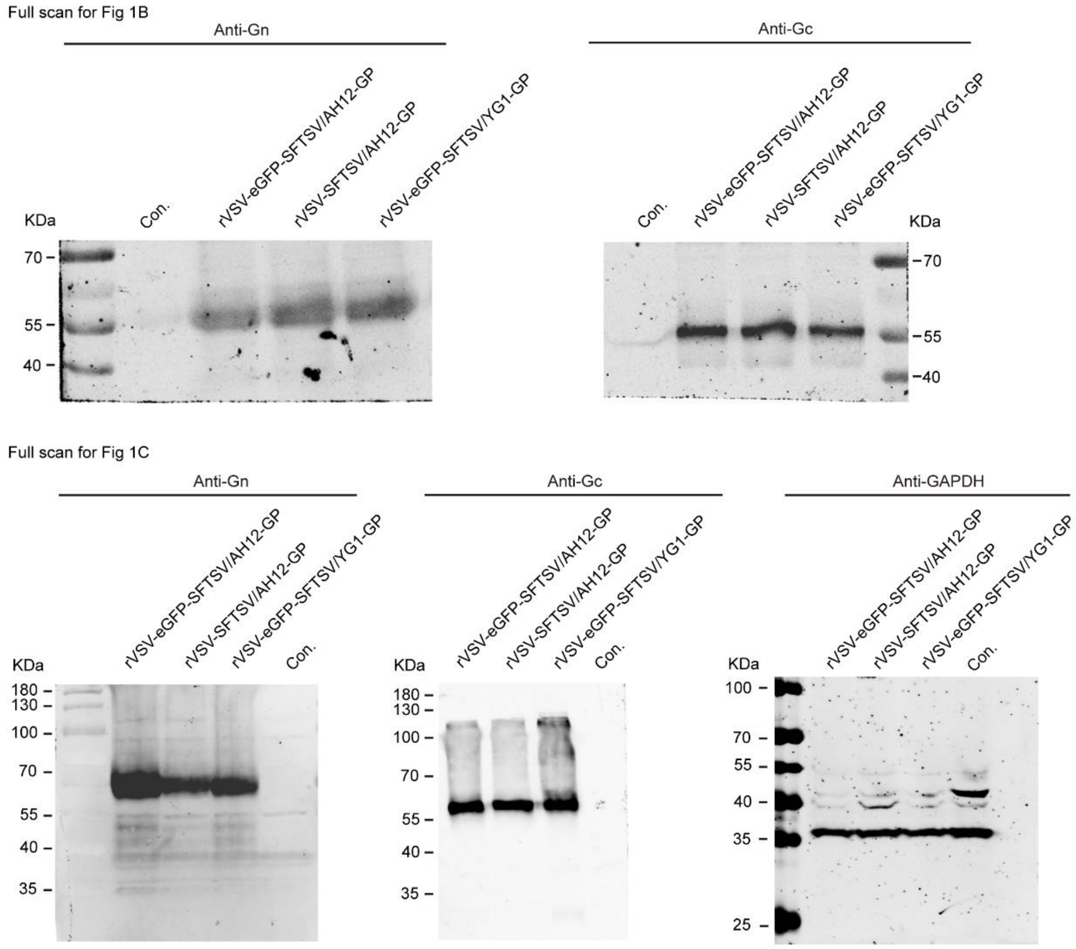

Supplementary figure 3. Full scan of Fig 1B

Supplementary Table 1

List of primers

Target	Primer sequence	Tm/℃
(i) Primers used for quantitative reverse transcription polymerase chain reaction		
SFTSV Wuhan strain	F: 5’-ATGGATAGCAGCGTCTCATCAAATC-3’<br>R: 5’-TGAGCGCACTGTATGAGGTAGGTAA-3’	60
β-Actin(mice)	F: 5’-GGCTGTATTCCCCTCCATCG-3’<br>R: 5’-CCAGTTGGTAACAATGCCATGT-3’	60
(ii) Primers used for genotyping of the interferon α/β receptor knockout (IFNAR$^{-/-}$) mouse		
IFNAR	F: 5’-GGTACTTTCCGAGCCGCTTG-3’<br>R: 5’-TTCTCCCGATGTAACTAGCCCAG-3’	64
（iii） Primers used for sequencing of recombinant viruses		
rVSV-G	F: 5’-TGAACAATCCCCGGTTTACT-3’<br>R: 5’-CCAGTTCTTACTATCCCACAT-3’	60
rVSV-SFTSV/AH12-GP<br>(rVSV-eGFP-SFTSV/AH12-GP)	F: 5’-CGCGGATCCGACAGCGGCCCTATCATCTGT-3’<br>R: 5’-CGCGGTACCGGCGGGGTAGCACTGGGGGTT-3’	68
rVSV-eGFP-SFTSV/YG1-GP	F: 5’-TGGGCAAAGTGGGCGAAATC-3’<br>R: 5’-TAGGCACGCTGCTGTGGTAGGT-3’	55
rVSV-eGFP-HRTV-GP	F: 5’-GTGCGAGCAGGAGTCTATCC-3’<br>R: 5’-CTCTCTCCACTCGGACCTGA-3’	60

Abbreviations: F, forward; VSV, vesicular stomatitis virus; R, reverse.
